# A 14‐year‐old in heart failure with multiple cardiomyopathy variants illustrates a role for signal‐to‐noise analysis in gene test re‐interpretation

**DOI:** 10.1002/ccr3.1920

**Published:** 2018-12-14

**Authors:** Patrick S. Connell, Aamir Jeewa, Debra L. Kearney, Hari Tunuguntla, Susan W. Denfield, Hugh D. Allen, Andrew P. Landstrom

**Affiliations:** ^1^ Department of Pediatrics, Section of Pediatric Cardiology Baylor College of Medicine Houston Texas; ^2^ Department of Pediatrics, Division of Pediatric Cardiology The Hospital for Sick Children Toronto Ontario Canada; ^3^ Department of Pathology & Immunology Baylor College of Medicine Houston Texas; ^4^ Division of Pediatric Cardiology, Department of Pediatrics Duke University School of Medicine Durham North Carolina

**Keywords:** cardiomyopathy, genetic testing, variant of unknown significance, whole exome sequencing

## Abstract

Variants of unknown significance in cardiomyopathic disease should be analyzed systematically based on the prevalence of the variant in the population compared to prevalence of disease, evidence that other variants in the gene are pathologic, consistency of prediction software on pathogenicity, and the current clinical consensus.

## 1 INTRODUCTION

As genetic testing becomes more expansive so has the identification of variants of unknown significance (VUSs). We present a child with features of multiple cardiomyopathic diseases hosting multiple VUSs. We use this case to demonstrate a methodology for variant re‐interpretation which may be applicable to genetic test interpretation for a variety of heritable diseases.

Cardiomyopathy is a primary disease of the myocardium resulting in cardiac dysfunction that cannot be attributed to other clinical causes.^1^ Different subtypes of cardiomyopathy include dilated (DCM), hypertrophic (HCM), arrhythmogenic right ventricular (ARVC), and left ventricular non‐compaction (LVNC) cardiomyopathies; each defined by morphologic, phenotypic, and genetic features that can be either common or unique to each subtype. DCM is a decreased ventricular function resulting from abnormal myocardial contraction and occurs in 0.57 per 100 000 children.^2^ While 35%‐40% of children with DCM have a gene mutation in one of many proteins associated with the cardiac sarcomere,^1^ the most common gene mutation associated with DCM occurs in a giant sarcomere protein called *TTN*‐encoded titin (TTN).^1^, ^3^, ^4^ *SYNE2*‐encoded spectrin repeat‐containing nuclear envelope 2 (SYNE2) encodes a nuclear envelope protein that links the nuclear envelope to the cellular cytoskeleton. Mutations in *SYNE2* are a rare cause of DCM.^1^ ARVC occurs when arrhythmias result in apoptosis of myocytes and replacement with fibro‐fatty tissue, predominantly of the right ventricle.^5^ With a prevalence of 1 in 5000‐10 000 individuals, ARVC results from mutations in genes encoding components of the cardiac desmosome, with mutations in *PKP2*‐encoded plakophilin 2 (PKP2) being most common.^6^, ^7^ In contrast, *PKP4*‐encoded plakophilin 4 (*PKP4*) is a rare cause of ARVC. LVNC is excessive trabeculations along the apex and free wall of the left ventricle associated with arrhythmias and loss of systolic function.^1^, ^9^ LVNC is less common than other cardiomyopathies, occurring in 0.12 of 100 000 children, and caused by mutations in large structural proteins such as TTN.^1^, ^4^

As clinical diagnostic modalities and genetic testing platforms have become more sensitive, overlap among cardiomyopathy subtypes are emerging, such as in LVNC, which coexists with DCM approximately 60% of the time.^1^ The same gene can also cause multiple cardiomyopathy subtypes, as seen in *TTN* (ARVC, DCM, LVNC), *LMNA* (DCM, HCM, restrictive cardiomyopathy), and *DSP* (ARVC, DCM).^1^, ^4^, ^5^ Further, while increasingly expansive clinical gene testing has allowed more sensitive detection of variants, there has been a rapid increase in the number of “variants of unknown significance” (VUSs) and increased uncertainty in test interpretation.^10^ Two phenomenon known to result in non‐Mendelian inheritance of genetic disease further complicate the analysis: (a) Compound heterozygosity whereby two different mutant alleles in the same gene can cause disease in a heterozygous state and (b) multigenic disease whereby multiple mutant alleles in different genes cause disease when each in isolation is insufficient. Here, we present a case of mixed cardiomyopathy, with pathologically confirmed characteristics of DCM, ARVC, and LVNC, in the context of multiple VUSs in *PKP2*,* PKP4*,* SYNE2*, and *TTN*. Given recent advances in variant interpretation, reanalysis of these findings suggests that none of these variants is likely to be pathogenic in isolation. We cannot exclude the possibility that a combination of these variants may contribute to disease development in a multigenic fashion.

## 2 METHODS

### 2.1 Clinical evaluation and commercial genetic testing

This research study was approved by the Baylor College of Medicine Institutional Review Board, and informed consent was obtained from the family. Family history was obtained including coroner records from the proband's father. Clinical evaluation involving history, physical exam, laboratory testing, radiography, electrocardiography, and echocardiography was conducted. Genetic testing consisting of the comprehensive cardiomyopathy gene panel was sent to John Welsh Cardiovascular Diagnostic Laboratory in the Texas Children's Hospital Heart Center (Houston, TX).

### 2.2 Re‐interpretation of genetic test findings

Minor allele frequency was evaluated using the Genome Aggregate Database (GnomAD).^11^ In silico pathogenicity modeling was conducted using PolyPhen‐2,^12^ Provean,^13^ Mutation Taster,^14^ and Align GVGD.^15^ Variants were searched in ClinVar to obtain up‐to‐date consensus diagnostic significance.^16^ Variants were then reclassified based on 2015 American College of Medical Genetics (ACMG) guidelines.^17^

## 3 RESULTS

### 3.1 Clinical presentation and course

**Figure 1:**
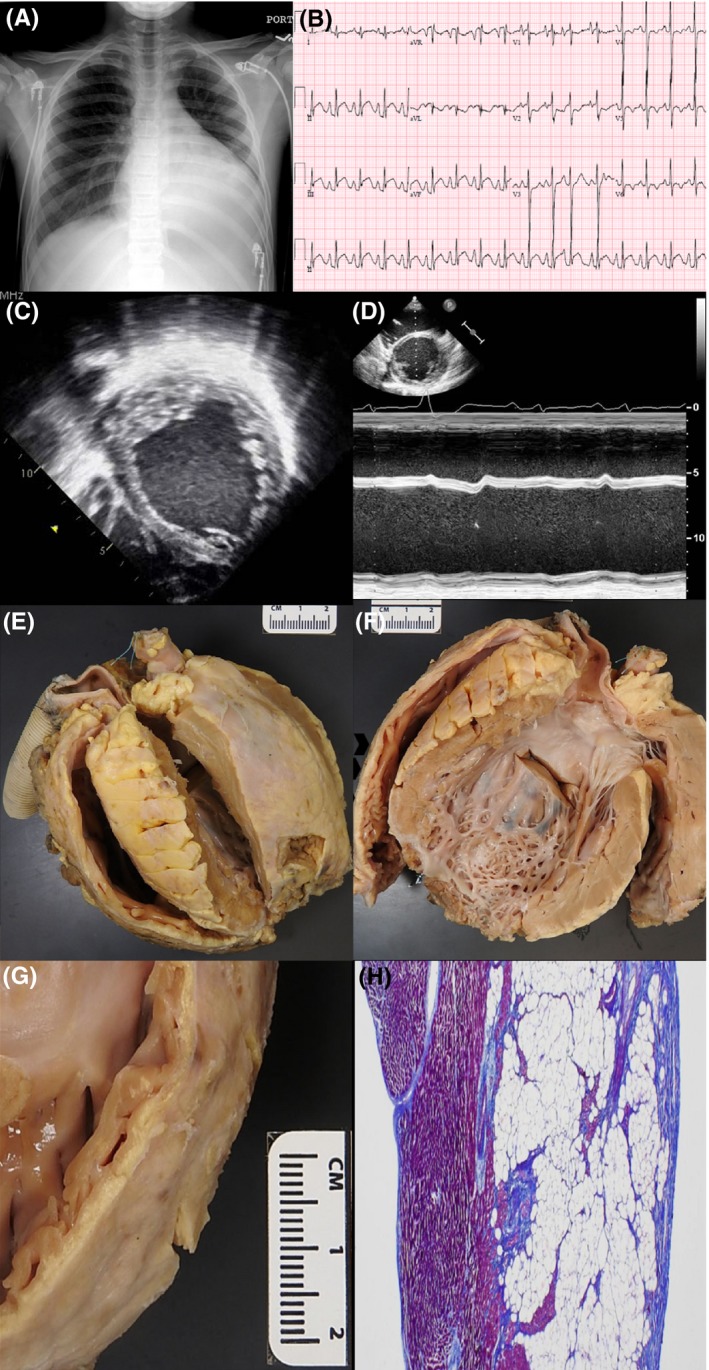
A, Chest X‐ray demonstrates cardiomegaly, pulmonary congestion, and left pleural effusion. B, ECG demonstrates sinus tachycardia with premature atrial complexes, biatrial enlargement, ST elevation in inferior leads, and T‐wave inversion in lateral leads. C and D, Echocardiogram demonstrates severely dilated left and right ventricles with prominent left ventricular trabeculations. E, Gross anatomy of explanted heart demonstrates dilated thin‐walled RV and dilated globular LV. F, LV chamber demonstrates severe dilation and a hypertrebeculated LV apex and free wall. Left ventricular assist device insertion site excision is seen at the apex. G and H, High‐resolution gross image and histologic view of the RV free wall shows fibro‐fatty infiltration of the myocardium with sparing of the subendocardium

A 14‐year‐old, previously healthy male presented to the emergency department with abdominal pain, emesis, and leg edema. His family history was notable for the sudden death of his father at age 58 while incarcerated. His autopsy was consistent with advanced coronary artery disease without features of cardiomyopathy. The patient's chest radiograph showed pulmonary congestion, left pleural effusion, and an enlarged cardiac silhouette (Figure 1A). Electrocardiogram showed sinus tachycardia with premature atrial complexes, biatrial enlargement, ST elevation in inferior leads, and T‐wave inversion in lateral leads (Figure 1B). BNP was elevated at 2280 pg/mL. Echocardiogram showed a four‐chambered heart with normal orientation and structure aside from a severely dilated left ventricle (LVDD 7.60 cm, *Z*‐score = 6.22) and severely depressed biventricular systolic dysfunction (FS 1.77%, EF by bullet method of 13%). Prominent LV trabeculations were noted (Figure 1C,D). Viral PCR for enterovirus and adenovirus was negative. There was no evidence of inflammatory heart disease by endomyocardial biopsy. Hemodynamic analyses showed right atrial mean pressure 23 mm Hg, right ventricular end diastolic pressure 20 mm Hg, mean pulmonary artery pressure 32 mm Hg, and mean pulmonary capillary wedge pressure 28 mm Hg.

Given the patient's sudden presentation in decompensated heart failure, there was a high suspicion for possible myocarditis despite the lack of inflammatory heart disease by biopsy. Therefore, the patient was treated with IVIG. Due to continued clinical decompensation on inotropic support with milrinone, a HeartMate II left ventricular assist device was placed and the patient eventually underwent cardiac transplantation. Gross anatomic and microscopic evaluation of the explanted heart showed left ventricular hypertrabeculation (Figure 1E,F). Although the trabeculations comprised less than 50% of the wall thickness, abundant apical trabeculations had been previously excised with placement of the left ventricular assist device, consistent with LVNC. In addition, extensive fibro‐fatty infiltration of right ventricle inflow and outflow sections with subendocardial sparing was found consistent with ARVC (Figure 1G,H). This evaluation did not demonstrate the inflammatory process expected in myocarditis. These findings were consistent with a final diagnosis of congenital cardiomyopathy, a combination of LVNC, ARVC, and DCM.

### 3.2 Cardiomyopathy gene panel testing

**Figure 2:**
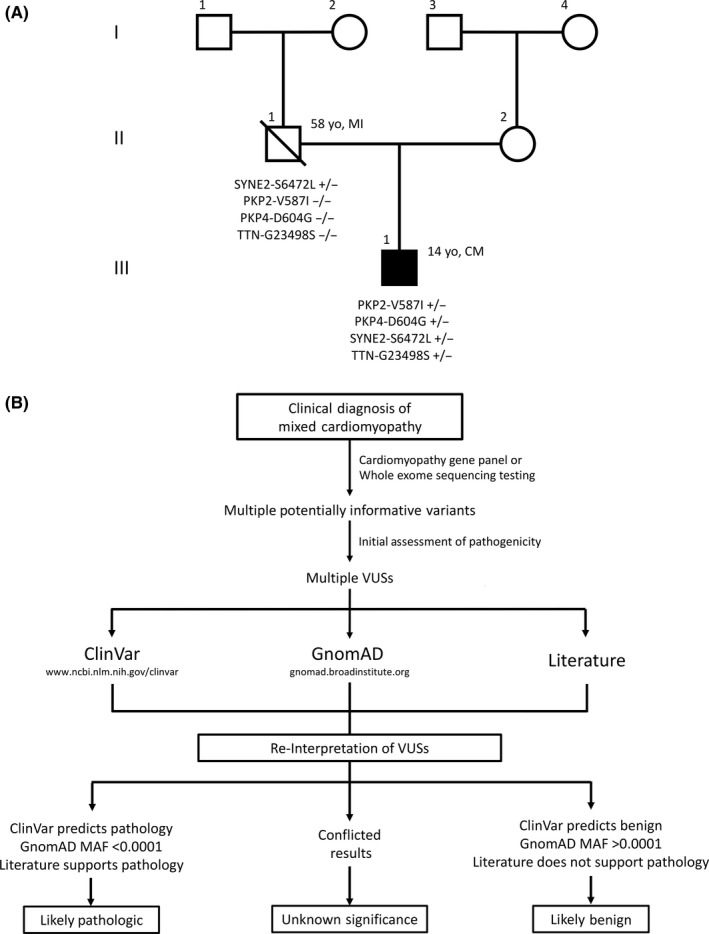
A, Pedigree showing the proband (III, 1). He does not have any known relatives with cardiomyopathy. His father (II, 1) passed at age 58 of myocardial infarction. MI, myocardial infarction; CM, cardiomyopathy. B, A flow diagram demonstrating the method by which multiple cardiomyopathy variants can be reanalyzed to determine their potential for monogenic disease pathogenicity

Based on the varied cardiomyopathy characteristics, next‐generation sequence analysis of a pan cardiomyopathy gene panel was obtained (John Welsh Cardiovascular Diagnostic Laboratory, Baylor College of Medicine, Houston, TX). This analysis identified four unique heterozygous missense variants PKP2‐V587I (c.1759G>A), PKP4‐D604G (c.1811A>G), SYNE2‐S6472L (c.19415C>T), and TTN‐G23498S (c.70492G>A) that were interpreted as VUSs with unclear diagnostic utility. Confirmatory postmortem testing of these variants in the proband's father was positive for SYNE2‐S6472L variant (Figure 2A). The proband's mother refused genetic testing and cardiac evaluation.

### 3.3 Reanalysis of genetic test results

In 2015, the American College of Medical Genetics (ACMG) published guidelines for interpreting genetic variants simultaneously with several studies examining healthy background variation in cardiomyopathy gene testing.^17^, ^18^ Together with the newly released Genome Aggregation Database (GnomAD), consisting of the exomes and genomes from 138 632 individuals, these guidelines provide an opportunity to reevaluate the diagnostic strength of the VUSs.^11^ In addition, a growing body of literature examining population‐based minor allele frequency (MAF) thresholds for “common” vs “rare” variants in cardiomyopathic disease has suggested that variants with MAF >1e‐4 are unlikely to have monogenic disease predisposition.^19^ Based on these findings, we developed a methodology for evaluation of cardiomyopathy associated VUSs (Figure 2B).

PKP2‐V587I has been previously associated with ARVC in a small number of patients in independent studies; however, this variant has a GnomAD minor allele frequency (MAF) of 2.4e‐3 and has also been found in healthy controls within cohort studies.^6^, ^20^, ^21^ Further, mutation prediction software did not consistently predict variant pathogenicity, and the consensus diagnostic significance from ClinVar was “benign to uncertain.” Based on ACMG criteria, PKP2‐V587I was reclassified as likely benign.

PKP4‐D604G has been identified in a limited series of ARVC patients without mutations in other known ARVC‐causing genes.^8^ With a GnomAD‐established MAF of 3.5e‐3, the frequency of this variant is higher among healthy individuals than in established ARVC cohorts. The affected amino acid which is highly conserved and predicted to be pathologic by PolyPhen‐2, Provean, Mutation Taster, and Align GVGD^8^, ^20^ was not described in ClinVar. Based on ACMG criteria, PKP4‐D604G was reclassified as uncertain significance.

The SYNE2‐S6472L variant has not been described in patients with ARVC, has a MAF of 2.6e‐3^1^, ^22^, and has mixed pathogenicity in an in silico prediction profile. The consensus diagnostic significance of this variant by ClinVar was “likely benign.” Based on ACMG criteria, SYNE2‐S6472L was reclassified as likely benign.

Lastly, the TTN‐G23498S variant is a missense mutation that has not been directly associated with cardiomyopathy. The majority of pathogenic *TTN* variants result in radical protein truncations, while TTN*‐*G23498S is a missense mutation. GnomAD‐established MAF was 2.3e‐4, and in silico prediction software inconsistently predicted pathogenicity. The consensus diagnostic significance of this variant by ClinVar was “uncertain significance.” Based on ACMG criteria, TTN‐G23498S was reclassified as likely benign. These findings are summarized in Table 1.

**Table 1:** Summary of genetic variants

Gene	DNA Change	Amino Acid Change	MAF	MAF Cutoff	PolyPhen‐2	Provean	Mutation Taster	Align GVGD	Clin Var	Associated CM	ACMG Analysis (Criteria)^a^
*PKP2*	c.1759G>A	V587I	2.4e‐3	Above	PD	N	DC	C25	LB‐U	ARVC	LB (BS1, BP6)
*PKP4*	c.1811A>G	D604G	3.5e‐3	Above	PD	D	DC	C65	N/A	ARVC	US (BS1, PP3)
*SYNE2*	c.19415C>T	S6472L	2.6e‐3	Above	PD	N	DC	C65	LB	DCM	LB (BS1, BP6)
*TTN*	c.70492G>A	G23498S	2.3e‐4	Above	B	D	PM	C55	US	DCM, ARVC, LVNC	LB (BS1, BP1)

ARVC, arrhythmogenic right ventricular cardiomyopathy; BP, benign strong; BP, benign supporting; CM, cardiomyopathy; D, deleterious; DC, disease‐causing; DCM, dilated cardiomyopathy; LB, likely benign; LB‐U, likely benign to uncertain; LVNC, left ventricular noncompaction; MAF, minor allele frequency; MAF cutoff, variant frequency compared to MAF rare variant threshold of 1e‐4; N, neutral; PD, probably damaging; PM, polymorphism; PP, pathologic supporting; US, uncertain significance.

a Criteria are based on 2015 American College of Medical Genetics (ACMG) guidelines.^17^

Based on these data sources, we conclude that all variants are unlikely to cause disease in isolation because (a) each variant existed at high background frequency and can be classified as “common genetic variation,” (b) inconsistent in silico pathogenicity predictions, and (c) absence of ClinVar consensus opinion in favor of pathogenicity. In addition, ACMG criteria reclassify the variants in *PKP2, SYNE2,* and *TTN* as likely benign.

## 4 DISCUSSION

Recent advances in genetic analysis allow clinicians to interrogate the genome of patients with cardiomyopathy to a degree not previously achievable. These analyses help uncover the underlying cause of cardiomyopathy, thus providing a foundation for genetic counseling for patients and their families. However, caution should be used when interpreting results generated by these methods because although they provide more sensitivity, they are also less specific than other diagnostic evaluations. This is especially true for VUSs, such as those presented here. While expert laboratories provide analysis of pathogenicity when reporting variants to clinicians, there is frequently discordance in labeling‐specific variants as pathogenic when comparing the findings from different laboratories.^23^ Therefore, it is vital that clinicians interpreting such reports in their clinical decision making understand how to interpret genetic reports and confirm their findings independently if necessary. Here, we report a 14‐year‐old with mixed cardiomyopathy phenotypes including three diseases that are traditionally believed to be discrete clinical entities (DCM, ARVC, and LVNC). Genetic testing showed four missense variants that provide a potential basis for the sequelae of cardiac disease present in this patient.

Following reanalysis, we conclude that none of these variants are likely to be the etiology of disease in isolation with little diagnostic relevance. For instance, approximately 1 in 400 individuals are predicted to carry the PKP2‐V587I MAF variant. This genetic background “noise” severely undercuts the possibility that this variant confers disease susceptibility. While robust studies regarding its prevalence are lacking in ARVC, conservative estimates place the prevalence at 1 in 5000 in the general population, with *PKP2* variants making up ~35% of all ARVC cases. Thus, the background genetic noise of this variant far exceeds the “signal” of disease pathogenicity, making it unlikely to cause disease in isolation.

However, an alternative, non‐Mendelian disease mechanism could be impacting our proband. Concurrent *PKP2* and *PKP4* heterozygous missense variants have previously been found in an ARVC patient,^20^ suggesting a potential synergistic dysfunction if both desmosome proteins are impacted. This proposed pathogenic mechanism, known as multigenic disease, occurs when two or more gene variants that do not result in clinical pathology alone occur along the same pathway or among proteins with a strong pleiotropic interaction, resulting in summative pathologic impact. While a possibility, multigenic disease is a challenge to conclusively prove and overall hard to replicate across multiple studies.^24^ Further, we cannot exclude the possibility that there is a novel monogenic cause of this child's cardiomyopathy in a gene locus that is yet unidentified. Ultimately, while multigenic effects from multiple genetic loci cannot be ruled out as a factor in the development of cardiomyopathy of our proband, we conclude that there is insufficient evidence for any single mutation to be solely disease causative. As a consequence of this reanalysis, confirmatory testing of these variants in clinically healthy family members is not indicated.

Bayes theorem suggests that genetic test interpretation should be viewed as a probability modifier of pretest probability and not a binary “positive” or “negative” finding. Stated another way, ARVC gene variants are more likely to be interpreted as pathogenic when coupled with a strong clinical diagnosis of ARVC in genes commonly found to be mutated in ARVC. Clinical heterogeneity, as in this case, demonstrates how pretest probability can be challenging to determine, even in the context of true disease. This also informs the interpretation of variants in genes that are rarely associated with disease, such as *PKP4*. Variants of this gene are less likely to cause disease even when found in affected patients because their low prevalence reduces their positive predictive value. Overall, improved clinical utility will depend on additional investigation into clinical population WES data to explore highly frequent VUSs that occur in cardiomyopathy patients. In addition, further functional genomic exploration is needed to experimentally validate whether VUSs are clinically meaningful. In the interim, it is important to analyze VUSs in the proper clinical context, especially when presenting this information to patients, their families, and when using these data to make clinical decisions.

## CONFLICT OF INTEREST

There are no conflict of interests.

## AUTHOR CONTRIBUTION

PC: collected and analyzed the case, performed genetic re‐analysis, drafted the initial manuscript, and reviewed and revised the manuscript. AJ, HT, SD: collected clinical data, reviewed and revised the manuscript. DK: provided pathology images, provided expert commentary on pathology images, reviewed and revised the manuscript. HA: led IRB approval process, provided clinical input, reviewed and revised the manuscript. AL: led IRB approval process, obtained informed consent, provided interpretation of the genetic testing and conclusion, reviewed, revised and supervised drafting of the manuscript. All authors approved the final manuscript as submitted and agree to be accountable for all aspects of the work.
